# Small vertebrates running on uneven terrain: a biomechanical study of two differently specialised lacertid lizards

**DOI:** 10.1038/s41598-019-53329-5

**Published:** 2019-11-14

**Authors:** François Druelle, Jana Goyens, Menelia Vasilopoulou-Kampitsi, Peter Aerts

**Affiliations:** 1 Laboratory for Functional Morphology, University of Antwerp, Antwerp, Belgium; 2 Department of Sport Sciences, University of Ghent, Ghent, Belgium

**Keywords:** Evolutionary ecology, Experimental evolution, Biomechanics

## Abstract

While running, small animals frequently encounter large terrain variations relative to their body size, therefore, terrain variations impose important functional demands on small animals. Nonetheless, we have previously observed in lizards that running specialists can maintain a surprisingly good running performance on very uneven terrains. The relatively large terrain variations are offset by their capacity for leg adjustability that ensures a ‘smooth ride’ of the centre of mass (CoM). The question as to how the effect of an uneven terrain on running performance and locomotor costs differs between species exhibiting diverse body build and locomotor specializations remains. We hypothesise that specialized runners with long hind limbs can cross uneven terrain more efficiently than specialized climbers with a dorso-ventrally flattened body and equally short fore and hind limbs. This study reports 3D kinematics using high-speed videos (325 Hz) to investigate leg adjustability and CoM movements in two lacertid lizards (*Acanthodactylus boskianus*, running specialist; *Podarcis muralis*, climbing specialist). We investigated these parameters while the animals were running on a level surface and over a custom-made uneven terrain. We analysed the CoM dynamics, we evaluated the fluctuations of the positive and negative mechanical energy, and we estimated the overall cost of transport. Firstly, the results reveal that the climbers ran at lower speeds on flat level terrain but had the same cost of transport as the runners. Secondly, contrary to the running specialists, the speed was lower and the energy expenditure higher in the climbing specialists while running on uneven terrain. While leg movements adjust to the substrates’ variations and enhance the stability of the CoM in the running specialist, this is not the case in the climbing specialist. Although their legs are kept more extended, the amplitude of movement does not change, resulting in an increase of the movement of the CoM and a decrease in locomotor efficiency. These results are discussed in light of the respective (micro-)habitat of these species and suggest that energy economy can also be an important factor for small vertebrates.

## Introduction

Locomotion requires mechanical work to counter inertia (and gravity when moving upwards) and to overcome resistive forces from the environment. Issues relating to substrate structure and organisation alter the locomotion of animals, and adaptations for ecologically-relevant ways of moving can be found in various aspects of the animal biological system^1–3^. The design of the limbs^4,5^, the type of gait^6^ and the posture^7^ can, therefore, influence locomotor performance and efficiency. Relative to their body size, small animals are more prone to encounter large terrain variations than larger animals do. Apart from the fact that their locomotor cost (J/kg/m) is already high compared to large animals^8–10^, terrain structure and organisation at the scale relevant to the animal may impose important additional energetic challenges in small animals. For instance, uneven terrain requires manoeuvring and intermittent running to bypass obstacles or, can require moving up and down along the running trajectory to cross obstacles. Therefore, running over uneven terrains will unavoidably result in both perturbations of the overall goal directed movement as well as higher costs.

Encountering large terrain variation relative to body size is a very common scenario for small lizards. Investigating the impact of an uneven substrate on the kinematics of running lizards is, therefore, essential to gain insight into the relationship between fitness and performance in an appropriate ecological context. Although previous studies have explored obstacle negotiation in lizards^11–14^, hardly anything is known about crossing extensive uneven terrains. In our previous study^15^, we made the observation that *Acanthodactylus boskianus*, a running specialist, is not only specifically adapted for high-speed running on an even, level surface, but is also able to maintain its high performance on structurally uneven terrain. The relatively large terrain variations were offset by important capacities in leg adjustability that ensured a ‘smooth ride’ for the centre of mass. Despite being a desert species, *A. boskianus* is also adapted to deal with large terrain variations at a scale relevant to its size. The question as to how the effect of an uneven terrain on running performance and locomotor costs differs between species exhibiting different locomotor specializations remains. In this context, and assuming that the functional demands imposed by natural environments (in terms of structure and organisation) are reflected in the locomotor system^2,3^, lizards with different morphologies should exhibit different locomotor performance. According to the physiological and biomechanical theory, the sprinters would benefit from a laterally compressed body, long hind limbs (with primarily long and slender zeugopods and autopods) and a more parasagittal running limb posture. This enables these lizards to take large strides and to reach high speeds. In contrast, the climbers would benefit from dorso-ventrally flattened bodies and strong, short equal fore and hind limbs with a sprawling posture to enable them to keep a close and firm contact with the substrate^1,16–21^.

Here, we compare locomotor performance and kinematics in a running specialist belonging to the Lacertidae family, *A. boskianus*, to a climbing specialist, the lacertid *Podarcis muralis*, when negotiating an uneven terrain. Both species are described as active foragers, i.e. species for which the locomotion accounts for a large portion of the energy budget (mean number of moves per minute: 2.01 and 3.05, respectively, and percentage of time moving: 28.80% and 20.54%^22^), but they are representative of different locomotor specializations. *P. muralis* is described as a specialized climber primarily seen as a rock-dwelling lizard, thus commonly encountering both highly uneven and vertical structures as well as flat terrains^23–25^. *A. boskianus* is considered a specialist in fast running and acceleration in an open desert environment^26–29^.

According to our previous results^15^, we presently hypothesise that the running costs (J/kg/m) should hardly be affected by the imposed terrain variations in *A. boskianus*. The anticipated good performance of the running specialist on the uneven terrain may be related to naturally occurring sand ridges in its (micro-)habitat. In the present study we also compare the centre of mass dynamics, limb behaviour and locomotor costs of *A. boskianus* with results for *P. muralis* when tested on the same terrain. Although the climbing specialist commonly lives in rocky and uneven environments, our experimental terrain should strongly perturbate the running performance of this species because its habitat commonly offers many hiding places that do not require running any great distance. Furthermore, their anatomy, i.e. a flattened body with short limbs (see above), allows it to maintain close and firm contact with the substrate, thus *P. muralis* are expected to follow the uneven substrate topology closely, leading to perturbations in their running mechanics. In this context, we hypothesise that, on the flat terrain, *A. boskianus* will show better locomotor performance and lower costs to those of *P. muralis*^17,30,31^. Furthermore, the latter should be more perturbated by the uneven terrain than the running specialist. The respective limb and CoM dynamics should result from the differences in limb length and design^4,32^ and from the respective ecologically-relevant escaping strategies of these species, i.e. running a great distance in *A. boskianus* and hiding as fast as possible in *P. muralis*.

## Methods

### Subject details

Seven adult male *A. boskianus* were obtained from a commercial dealer (Amfibia, Antwerpen, Belgium) and seven adult male *P. muralis* were collected using hand foraging techniques in the wild (Mechelen, Belgium; the *P. muralis* individuals were released in their natural environment after the experiments). All animal care and experimental procedures were carried out in accordance with the regulations and guidelines of the University of Antwerp. The present protocol was approved by the ethical committee of the University of Antwerp (ECD-dossier 2013-76).

### Experimental protocol and acquisition of data

We constructed an adjustable racetrack including a central part that could remain flat (control) or be covered with hemi-spheres (uneven terrain). The hemi-spheres were 25 mm high, i.e. equal to ≈0.4 times snout vent length of our animal sample (63.95 ± 3.18 mm in *A. boskianus* and 61.26 ± 3.19 mm in *P. muralis*). We painted the flat and uneven terrains with adhesive paint and sand was additionally spread and glued to the surface. This significantly increased the roughness of the substrates to enable the animals to run at top speed.

The experiments took place in the morning in November 2017 for *A. boskianus* and in April 2018 for *P. muralis*. All the animals were first kept in an incubator set at 37 °C for *A. boskianus* and 30 °C for *P. muralis* to optimise their respective locomotor performance^26,28^. For each individual, 15 anatomical parts were marked with white using water based paint: top of the snout, back of the head, side of the head, shoulder, mid-trunk, hip, mid-tail, knees, proximal part of the feet, elbows and proximal part of the hands. During a 3-week period, we tested each lizard randomly on each substrate every day. The lizards were encouraged to run along the racetrack by means of hand chasing and one or two consecutive trials were performed per substrate [a minimum of 30 minutes rest time (in the incubator) between the different per-substrate trials was ensured]. We recorded the running animals with four synchronized high-speed digital video cameras operating at 325 frames.s^−1^ and 1/800 shutter speed (© 2018 NorPix Inc., system 10 GigE Vision, 1920 × 1080). The cameras were positioned perpendicular to the runway, at the top and in diagonal for increasing the accuracy of the 3D reconstruction (see Figure A in Supplementary Material). Calibration was performed using a custom-made calibrated construction (477 × 143 × 96 mm) on which 40 dots were digitized. After the recording, the digitization of the body markers was performed manually frame-by-frame using Matlab (R2019a) and the *DLTdv5* application developed by the T. Hedrick lab^33^. A strong selection criterion was applied on the selection of the sequences to be digitized. Sequences were considered appropriate when the running individuals were crossing the substrate in a straight line and at a constant speed. This resulted in 61 strides analyzed for *A. boskianus* and 51 for *P. muralis*. Further information about the present experimental protocol can be found in our previous paper^15^.

### Locomotion analysis

On the raw data (digitized markers), we first applied a fourth order low-pass Butterworth filter with a cut-off frequency of 60 Hz. This is well above the mean stride frequency in our study (mean frequency = 11.74 Hz in *A. boskianus* and 11.69 Hz in *P. muralis*). Second, a general filter using a piecewise cubic spline interpolation method was applied for missing data. For instance, the very fast movement of the limbs during the swing phase sometimes made few dots impossible to digitize correctly. In these occasional cases, we kept the running sequence with the few missing dots (usually 1–5 frames). If more than one third of the frames was missing, we removed the complete stride from the dataset. We then estimated the position of the body centre of mass (CoM) based on the dissections of three *A. boskianus* cadavers^15^ and one *P. muralis* cadaver. After freezing and segmenting the body, the body parts were subsequently weighed on a micro balance (MT5 Mettler Toledo, Greifensee, Switzerland; precision: 0.01 mg), and each marker was provided with a percentage of the total body mass (the limb CoMs are estimated at the knees and elbows). The weighted arithmetic mean of all markers enabled us to calculate the instantaneous position of the CoM in all digitised frames. We corrected the height of the CoM for substrate height by substracting 25 mm from CoM height on the uneven terrain (i.e. the radius of the hemi-spheres). In our sample, the average position of the CoM was estimated to be 23.9 ± 1.9% of the trunk from the hip joint for *A. boskianus* and 23.7 ± 4.9% for *P. muralis*. The estimated trajectory of the CoM from the slope of the regression line in the XY-plane allowed us to recalculate the global frame of reference using a rotation matrix, with an X-axis aligned with the direction of motion, and the Y-axis perpendicular to the X-axis in lateral direction; the Z-axis is aligned with the gravity vector.

Morphometrics and body movements were used to determine the instantaneous mechanical energy of each body segment (head, proximal trunk, mid-trunk, distal trunk and tail) over a stride period:

$${E}_{si}=mgZ+\frac{m({\dot{Z}}^{2}+{\dot{X}}^{2}+{\dot{Y}}^{2})}{2}+\frac{I(\dot{y}{a}^{2}+\dot{p}{i}^{2})}{2}$$

Where *m* is the mass of the segment *si*, *g* is the gravitational acceleration (9.81 m.s^−2^), *Z* is the instantaneous height of the CoM of the segment considered (the segment CoM is estimated from the different markers), $\dot{Z}$, $\dot{X}$ *and* $\dot{Y}$ are the linear velocity of the segment CoM, $\dot{y}a$ and $\dot{p}i$ are the angular velocity of the segment *si* in the frontal and sagittal plane, respectively; note that the roll rotation is not included in the calculations as it is expected to be minimal comparing to the yaw and pitch. *I* is the inertia of the segment *si* and is estimated using the moment of inertia calculation for a uniform rod, as follows:

$${I}_{si}=\frac{m{L}^{2}}{12}$$

Where *L* is the length of the segment *si*. Each limb was considered as a point mass at the level of the elbow or knee and the instantaneous mechanical energy was calculated as follows:

$${E}_{pi}=mgZ+\frac{m({\dot{Z}}^{2}+{\dot{X}}^{2}+{\dot{Y}}^{2})}{2}$$

The total instantaneous minimal energy is calculated as the sum of all *E*_*si*_ and *E*_*pi*_ and the time differential of the total energy yields the instantaneous power during the stride. The integral of the positive power allows us to calculate the average positive work and the integral of the negative power allows us to calculate the average negative work over a stride.

The overall efficiency of the muscles depends on their contractile properties as well as their elastic components. Although the elastic components stretched during the preceding phase of negative work may increase the efficiency of the muscles, the maximal efficiency of the conversion of chemical energy into the positive mechanical work is approximately 25% in animals^34–36^. It has also been shown that large animals should benefit more from elastic energy savings than smaller animals^5,35^. Therefore, in the present study, we are assuming that muscles can perform positive work with a maximal efficiency of 25%. We therefore estimated the energy cost of transport from the sum of the positive work times 4 and the negative work times 1.

### Statistical analysis

#### Assessing morphological differences between species

Comparisons in morphometrics (body mass and segment lengths) were conducted between both species using exact Permutation tests for independent samples. In this context, the statistical unit is the individual and permutations are an appropriate test for the small sample size (n = 7).

#### Assessing kinematic differences among and within species

In the present protocol, a strong selection criterion had been previously performed on the running sequences (see previous). Each selected stride comes from a different running sequence, thus ensuring stride independence. In addition, using dimensionless quantities is a way to control for potential random effects related to individuals because we expect individual differences in running kinematics to be related to size. Hence, we have considered the strides as our experimental units and the strides are compared, on the one hand, across speed and species on the control substrate (a), and on the other hand, across speed and substrates within species (b). All kinematic data were log_10_-transformed before analysis in order to ensure normality and homoscedascity assumptions.

- *Between species on the control substrate* Using analyses of variance (ANOVA), we first tested for differences between species in mean speed and dimensionless speed [assessed using the Froude Number $\frac{{v}^{2}}{l\times g}$; where *v* is the stride average speed, *l* is the length of the tibia of the individual considered^15,37^, and *g* is the gravitational acceleration (9.81 m.s^−2^)]. Second, a set of covariance analyses (ANCOVAs) were performed on different response variables including species as factor and dimensionless speed as a covariate. The response variables tested are: the dimensionless spatio-temporal parameters [dimensionless stride length (stride length divided by tibia length), dimensionless stride frequency (squared frequency divided by tibia length times *g*) and duty factor (proportion of stance phase relative to stride duration)], the amplitude of CoM and foot displacements on the Y-axis (lateral) and Z-axis (vertical), the relative average position of the foot in the 3 planes, and the relative height at which the CoM is maintained.
- *Within species between substrates* The same statistical tests were performed but the substrate was included as a factor instead of the species in the ANCOVAs.

In addition, we compared the average cost of transport between species and substrates using ANOVAs. We also compared the slopes of the linear models between the cost of transport and absolute speed using the “lsmeans” package in R. All the statistical analyses were performed using R (version 3.3.2), but the permutation tests were performed using StatXact (version 3.1). The significance level was set at *P* < 0.05.

## Results and Discussion

### Morphological features associated to running and climbing skills in Lacertidae

**Figure 1:**
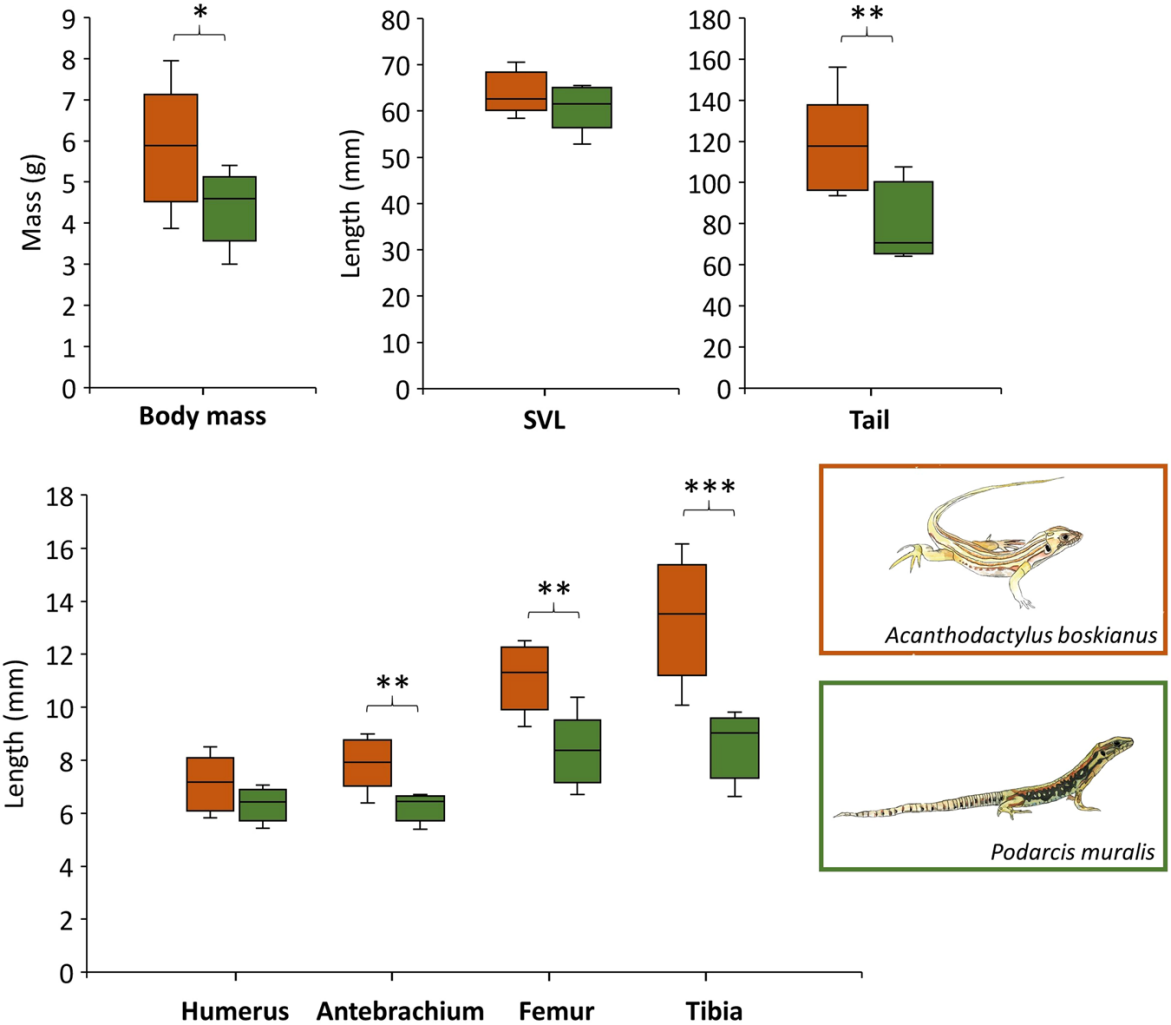
Comparisons of the measured morphological features between the running and climbing specialists. *A. boskianus* are in orange and *P. muralis* are in green. Symbol significance: *P < 0.05, **P < 0.01, ***P < 0.001. Lizard drawings are from Menelia Vasilopoulou-Kampitsi.

Figure 1 shows the morphological differences between *A. boskianus* and *P. muralis*. These differences can be related to their respective running and climbing skills. While the snout-vent length is not different between both species, *A. boskianus* has longer hind limbs (femur + tibia) than *P. muralis* (independent Permutation test = 3.062; *P* = 0.0006; Table A in Supplementary material). The mass of the hind limbs is more than 2 times larger than the mass of the forelimbs in *A. boskianus* (5.5 g *vs* 2.2 g), while fore- and hind limbs masses are almost equal in *P. muralis* (1.8 g *vs* 2.3 g). Both species exhibit longer hind limbs than forelimbs (*A. boskianus*: paired Permutation test = 2.551; *P = *0.0156; *P. muralis*: paired Permutation test = 2.514; *P* = 0.0156), but the difference between fore- and hind limb lengths is significantly larger in *A. boskianus* than in *P. muralis* (independent Permutation test = 2.806; *P* = 0.0012). The long hind limbs of *A. boskianus* relative to the forelimbs may enhance their running capacities, while the small difference in fore- and hind limb lengths in *P. muralis* certainly enhances their climbing skills^21^.

### Kinematic differences between runners and climbers when running on level surface

**Figure 2:**
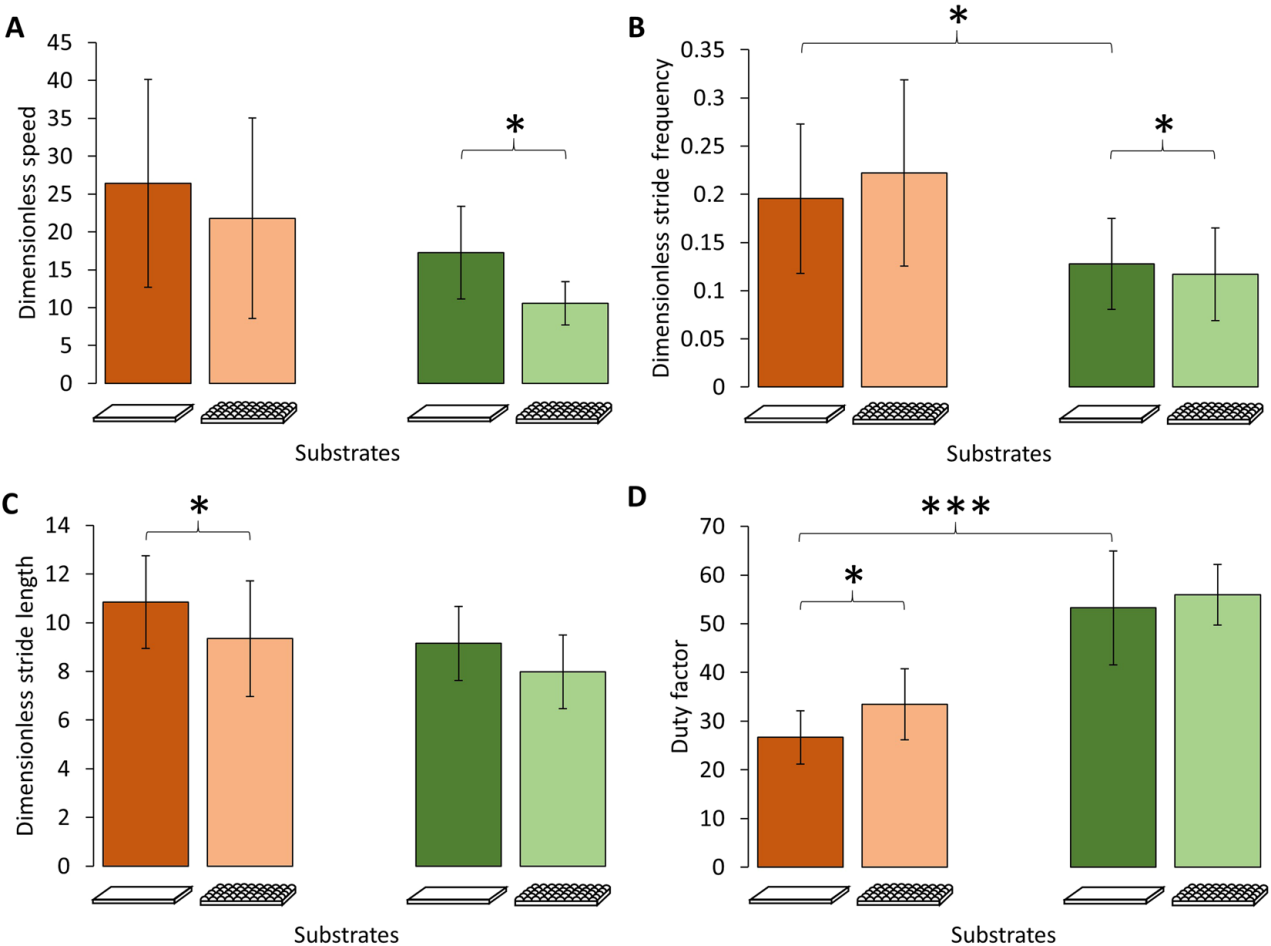
Average dimensionless speed (**A**) and spatio-temporal parameters calculated for each species and for each substrate: Dimensionless stride frequency (**B**), dimensionless stride length (**C**), duty factor (**D**). The brown colour is for *A. boskianus*, the green colour is for *P. muralis*. Within each species, darker bars represent the control (flat surface), lighter bars represent the uneven terrain (i.e. hemi-spheres). Error bars show standard deviations. Symbol significance: *P < 0.05, **P < 0.01, ***P < 0.001.

**Figure 3:**
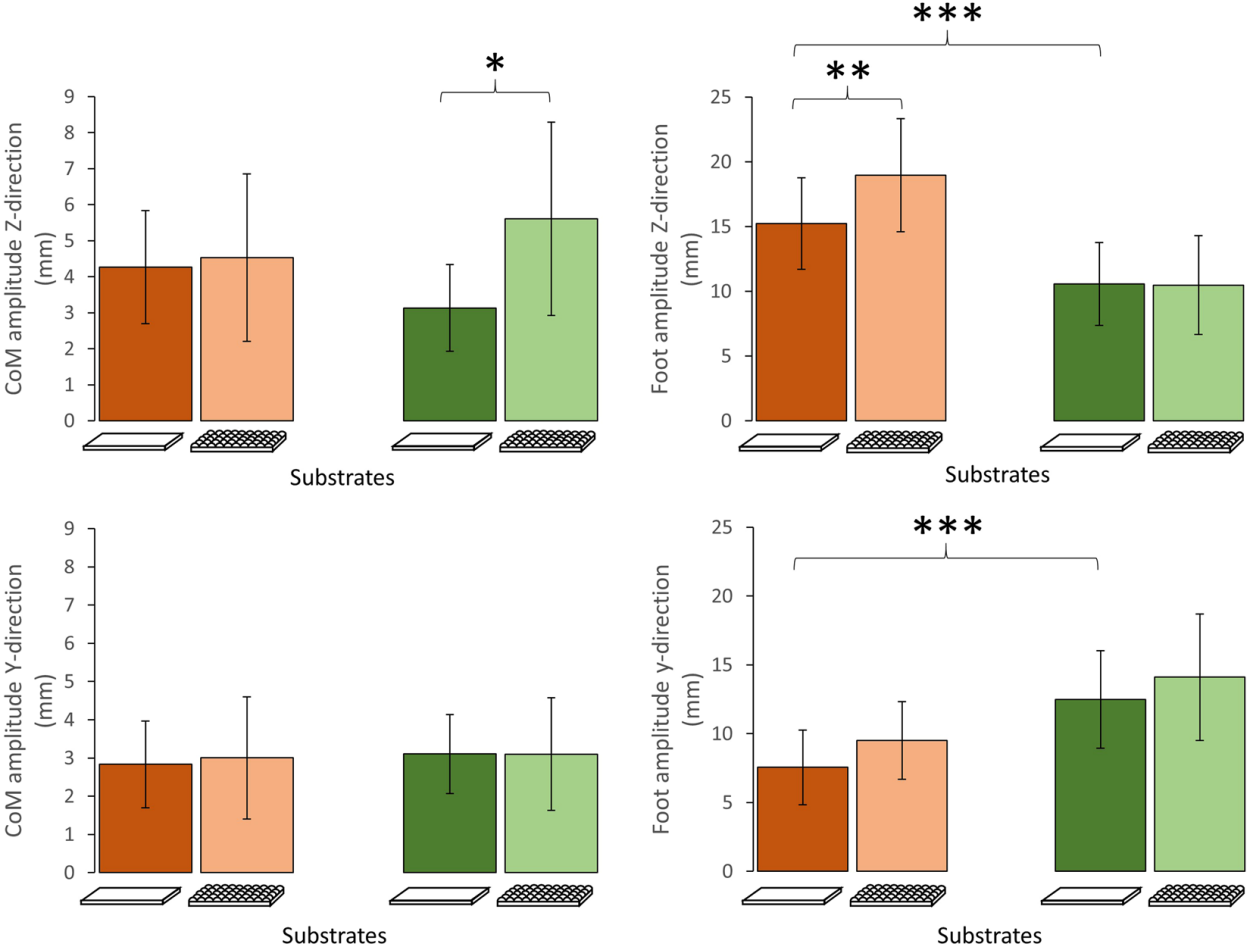
Average amplitudes of the CoM and foot displacements in the Y- and Z-directions. See Fig. 1 for symbol significance.

**Figure 4:**
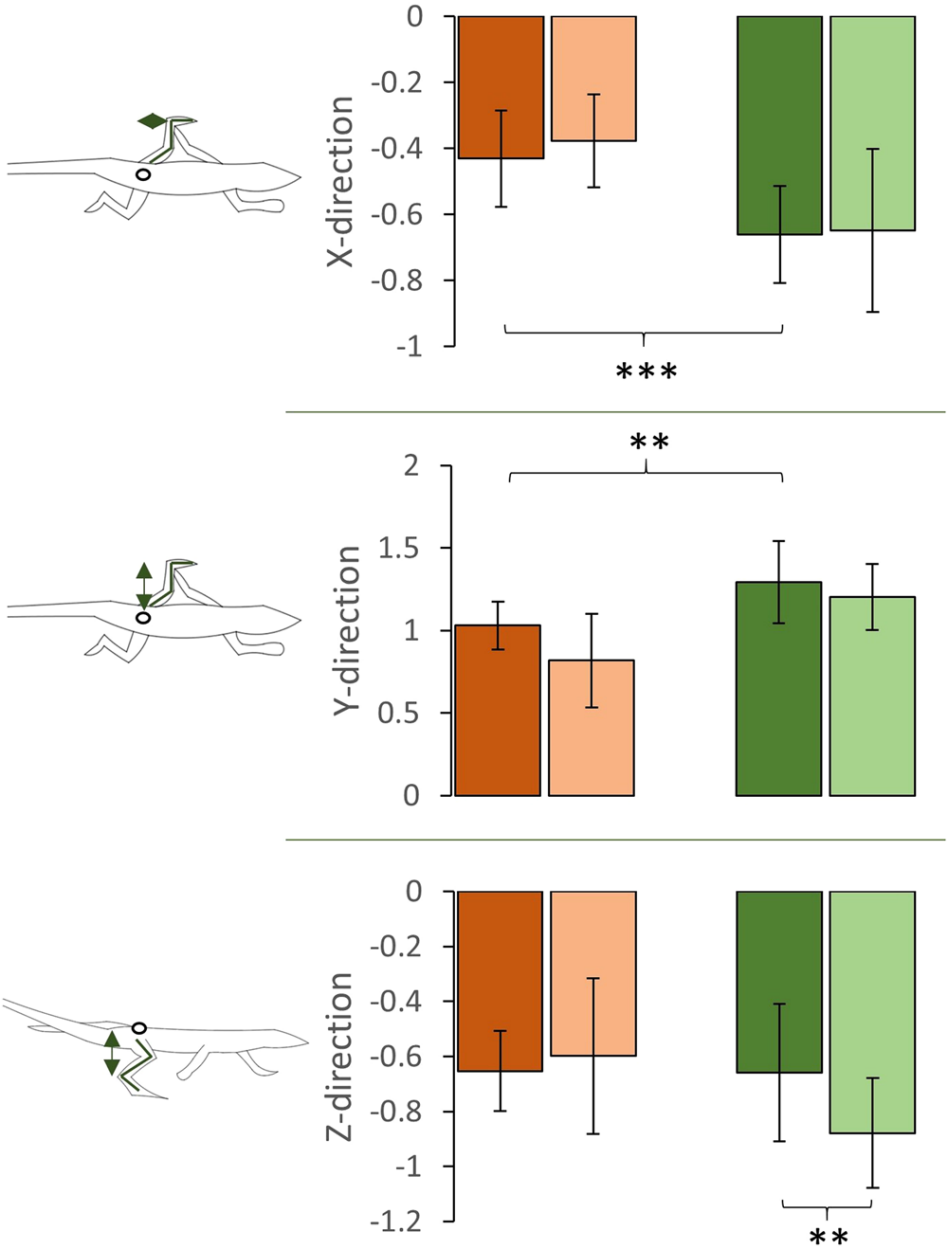
Average position of the foot relative to the hip per stride and corrected for size in the three planes of movement. See Fig. 1 for colour and symbol significance.

According to the trade-off hypothesis, being a specialist in one locomotor mode should impair performance in other modes^17,21,30,31^. Table 1 shows the average spatio-temporal parameters for a running specialist (*A. boskianus*) and a climbing specialist (*P. muralis*) when running on a flat/even substrate. *A. boskianus* run significantly faster than *P. muralis* (ANOVA F = 12.89, *P* = 0.0008). After correcting for size effects, there is no significant difference in the average speed between *A. boskianus* and *P. muralis* (ANOVA F = 2.395, *P* = 0.128; Fig. 2A). Correcting for size and speed effects, *A. boskianus* exhibits a higher stride frequency (ANCOVA F = 4.548, *P* = 0.0382; Fig. 2B) and a lower duty factor (ANCOVA F = 68.84, *P* < 0.0001; Fig. 2D) than *P. muralis*. The amplitude of the upward displacements of the foot (i.e. the foot clearance) is larger in *A. boskianus* (ANCOVA F = 15.53, *P* = 0.0003), while the lateral displacements of the foot are larger in *P. muralis* (ANCOVA F = 16.44, *P = *0.0002; Fig. 3). On average, *P. muralis* places its feet further, laterally, from the hip (ANCOVA F = 12.65, *P = *0.0009), i.e. the posture is relatively more sprawled, and the same happens in the fore-aft direction (ANCOVA F = 13.01, *P = *0.0007), i.e. the foot is more retracted in *P. muralis* (Fig. 4). There is no difference in the amplitude of CoM translation in the lateral and upward directions on the flat terrain. However, the CoM is maintained at a significantly lower height in *P. muralis* compared to *A. boskianus* (9.21 ± 3.03 mm and 17.15 ± 3.66 mm, respectively; ANCOVA F = 43.74, *P* < 0.0001). To sum up, *A. boskianus* use more parasagittal hind limb postures with a larger foot clearance, exhibit lower duty factor, higher stride frequency and keep the CoM relatively higher than *P. muralis*. These specificities thus emerge in *A. boskianus* which is a fast runner in general and a better sprinter than *P. muralis* on level surface. *P. muralis* run with a CoM very close to the surface, which is advantageous for balance in lizards that climb vertical surfaces^21^, while *A. boskianus* keep the CoM higher, avoiding touching the substrate and providing space for parasagittal limb displacements. In this way, *A. boskianus* run much faster, as observed in lizards living in open habitat^38^.

**Table 1:** Mean ± SD for spatio-temporal parameters.

	*A. boskianus*				*P. muralis*			
	Flat (control)		Uneven		Flat (control)		Uneven	
	Mean	SD	Mean	SD	Mean	SD	Mean	SD
Speed (m.s^−1^)	1.72	0.48	1.56	0.45	1.16	0.27	0.92	0.15
Stride frequency (Hz)	11.74	2.52	12.24	2.89	11.69	2.29	11.19	2.46
Duty factor (%)	26.67	5.49	33.43	7.28	53.25	11.66	55.97	6.22
Stride length (mm)	141.18	20.6	122.47	23.21	77.09	16.13	67.34	11.97

### Impact of running over an uneven terrain for a running specialist

**Figure 5:**
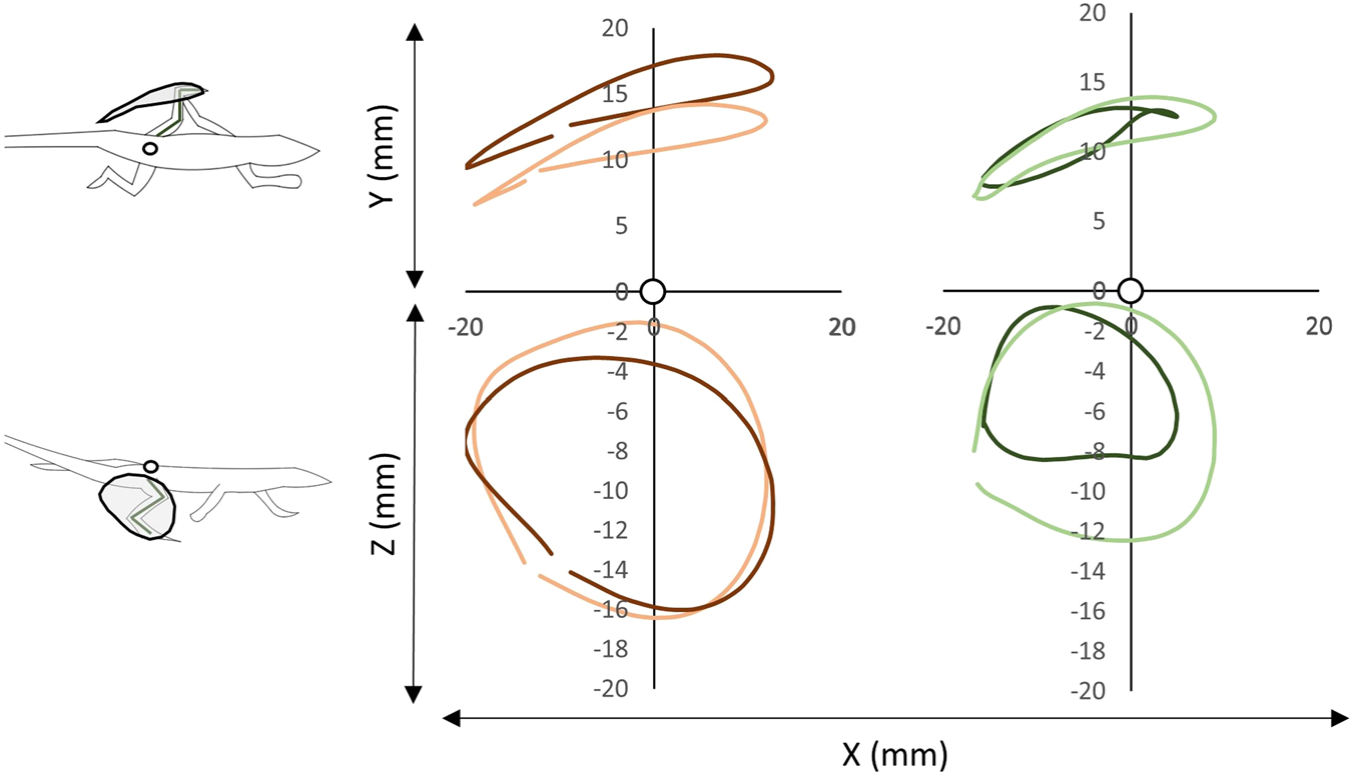
Mean trajectory of the foot movement relative to the hip on the sagittal (Z) and frontal (Y) planes (note that the orbits are not corrected for size). The hip is represented by a white circle. The orange colour is for *A. boskianus* (adapted from^15^), the green colour is for *P. muralis*. Within each species, darker orbits represent the control (flat surface), lighter orbits represent the uneven terrain (i.e. hemi-spheres).

According to previous research^15^, the small fast running specialist *A. boskianus*, is able to cope with complex substrates without there being any impact on sprint speed (Fig. 2). Although the stride length decreases (141 mm *vs* 122 mm; ANCOVA *F* = 4.075, *P* = 0.0482) and the duty factor increases (27% *vs* 33%; ANCOVA F = 9.134, *P* = 0.004), the average speed is not significantly impaired in this context. The orbit characterizing the movement of the foot relative to the hip also remains similar across the substrates in the sagittal plane (the XZ-plane) and in the frontal plane (the XY-plane; Fig. 5). We observed a significant increase in the amplitude of the foot clearance (ANCOVA F = 10.655, *P* = 0.0018; see Fig. 3) and the height at which the CoM is maintained decreases significantly (17.15 ± 3.66 mm *vs* 12.44 ± 3.28 mm; ANCOVA F = 16.565, *P = *0.0001). In general, the complex terrain impacts few kinematic aspects of *A. boskianus* (Figs 3 and 4). The changes occur mainly at the level of the legs that adjust instantaneously to the substrate variations through larger amplitudes of the foot clearance. This enables *A. boskianus* to keep the trajectory, as well as the movement amplitude, of the CoM stable.

### Impact of running over an uneven terrain for a climbing specialist

Contrary to *A. boskianus*, the average speed when negotiating the uneven terrain decreases significantly in *P. muralis* (1.16 ± 0.27 m.s^−1^ *vs* 0.92 ± 0.15 m.s^−1^; ANOVA *F* = 5.346, *P* = 0.025; Fig. 2). The stride frequency also decreases significantly (11.69 ± 2 Hz *vs* 11.19 ± 2 Hz; ANCOVA F = 9.148, *P* = 0.004; Fig. 2). Although the amplitude of the foot movements does not change on the uneven terrain, the centre of the orbit of the foot movement shifts downwards on the sagittal plane (ANCOVA F = 7.67, *P = *0.008; Figs 4 and 5); it does not change in the frontal and transversal planes. The CoM translation in the Z-direction increases (ANCOVA *F* = 5.09, *P* = 0.029) and the relative height at which the CoM is maintained decreases significantly (9.21 ± 3 mm *vs* 6.22 ± 2 mm; ANCOVA F = 7.732, *P = *0.0078).

**Figure 6:**
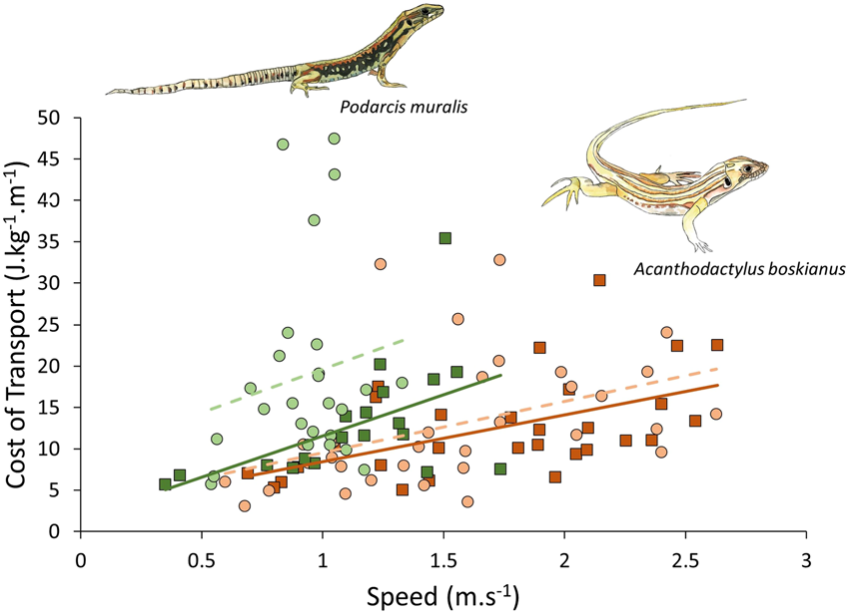
Relationship between speed and cost of transport (estimated from the fluctuations of the minimal mechanical energy) in *P. muralis* (in green) and *A. boskianus* (in orange). Squares indicate the strides performed on the flat (control) substrate and the solid lines represent the respective linear models, circles indicate the strides performed on the uneven substrates and the dashed lines represent the linear models.

### Costs of transport in running and climbing specialists

**Figure 7:**
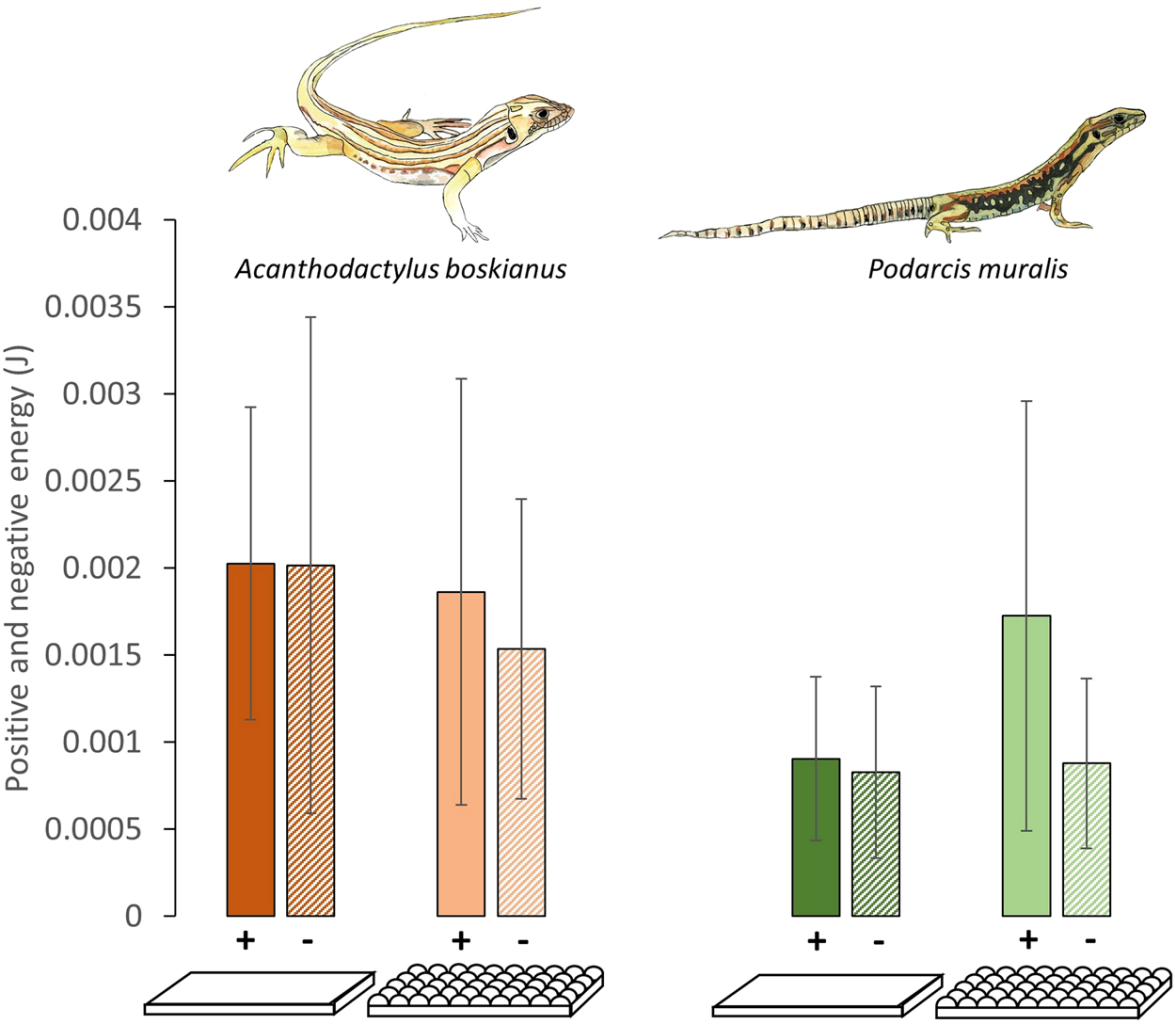
Positive and negative minimal mechanical energy in *A. boskianus* and *P. muralis* on the control and complex terrain. The solid colour represents the positive energy (+) and the diagonal lines represent the negative energy (−).

The cost of transport does not differ between the two species when running on a flat substrate (ANOVA F = 0.029, *P* = 0.87), however *A. boskianus* still run on average 50% faster than *P. muralis*. The substrate type (flat or uneven) does not impact the cost of transport in *A. boskianus* which supports the hypothesis that the morphology of *A. boskianus* is strongly adapted for fast running^27,29^. When these animals encounter large terrain variations relative to leg length, they can continue to minimise the energy costs related to running. In *A. boskianus*, leg movements adjust to the substrates’ variations, enhancing the stability of their CoM^15^. On the contrary, the complex terrain provokes a significant increase in the cost of transport in *P. muralis* (ANOVA F = 4.445, *P* = 0.041; Fig. 6) but the relationship between the cost of transport and speed is not impacted, i.e. there is no significant difference between the slopes of the regression lines among and within species. The general increase in the cost of transport in the climbing specialist *P. muralis* is mainly related to an increase of the positive external energy (Fig. 7). Although it can keep the energy costs associated with running at a low level on an even terrain, the costs increase significantly when the demands of the terrain become too high. In the context of the present uneven terrain, the running performance of *P. muralis* is strongly affected and the locomotor economy is lost.

Overall, our results show that runners and climbers have the same cost of transport on level terrain, although climbers run at a slower speed. Contrary to our hypothesis, the cost of transport is not higher in the climbers running on a level surface. Nevertheless, it is possible that the lower running speed of climbers allows them to maintain low energy costs (the slope between speed and cost of transport does not differ between the two species, but if *P. muralis* was able to run faster, Fig. 6 suggests even higher costs relative to *A. boskianus*). Thus, the morphological features associated with climbing impair sprint speed, as there is no difference in dimensionless speed. As anticipated, on the one hand, the uneven terrain has no influence on the average speed and the cost of transport in the running specialist, *A. boskianus*. On the other hand, the climber, *P. muralis*, encounters many more difficulties when negotiating uneven terrain. Indeed, speed and energy expenditure are impaired in *P. muralis* running on uneven terrain. In general, their legs are kept more extended, but the amplitude of movement does not change. Hence, leg movements do not adjust to the terrain as observed in *A. boskianus*, resulting in an increase of the movement of the CoM and a decrease in locomotor efficiency.

Movement is obviously related to muscle effort, and it can be expected that the maximal locomotor power output will be limited by the force that can be generated by the muscles. Some authors have argued that small animals (mammals and reptiles) do not rely on elastic energy mechanisms for locomotion, thus they exhibit important metabolic costs^27,35^. Although sprawled leg postures should increase the required muscle forces^32,39,40^, our results suggest that, overall, the costs of transport associated with running are similar in two lizard species adopting either more parasagittal leg postures (*A. boskianus*) or more sprawled postures (*P. muralis*). However, we have observed that the costs of transport are larger in *P. muralis* than in *A. boskianus* when crossing uneven terrain. The necessity to limit metabolic costs may be less important in fast climbers than in fast runners because climbers from rocky environments primarily rely on explosive power generation in order to find shelter rapidly within close proximity. This is indeed a typical behavioural strategy of *P. muralis*^25^. On the other hand, small running specialists such as *A. boskianus* definitely need endurance too, in order to escape to the nearest hiding place, which can be located at a distance, certainly for a desert species such as *A. boskianus*. As a result, they need to be able to keep the energy costs associated with fast locomotion at a low level; when targeted by a predator, making a stop in the middle of the pathway is not an option. *A. boskianus* can minimize the energetic challenges imposed by uneven terrain by limiting the movement amplitude of the CoM. Our study, therefore, supports the assumption that locomotor economy is optimized in accordance to the ecological relevance^35^.

## Conclusion

The capacity to negotiate uneven terrain at the scale of the animal size is not a common capacity shared by lizards in general. The climbing specialist tested in this study displays the lowest performance on uneven terrain. Saxicolous habitats are the primary niche of *P. muralis*, and it certainly poses many opportunities for hiding and escaping. In this way the obstacles and vertical substrates that have to be dealt with are commonly much larger than the size of these lizards. Our finding of the lower velocity and a higher energy cost on the uneven terrain for *P. muralis* compared to *A. boskianus*, support the theory that the former uses a behavioural strategy of swiftly escaping to a close hiding place when confronted with danger. For them, short running burst can be very effective. The running specialist *A. boskianus*, on the other hand, presumably runs away rapidly over long(er) distances under similar circumstances. This can, again, be linked to its specific structural microhabitat. *A. boskianus* lizards live in open environments such as deserts, where hiding spots can be located a long distance away. The specific structural microhabitat found in the desert may resemble most closely the uneven terrain in our experiments because on sandy substrates, sand ridges are often present, as a result of complex interactions between flowing sand masses and wind. This microstructure of a substrate that is very flat on a larger scale, may challenge small lizards such as *A. boskianus* in a very similar way as the uneven terrain in our experiments. This could explain why they perform so well on this substrate, both in terms of velocity and energy expenditure. Our study, therefore, supports the hypothesis that microhabitats impose functional demands that species are adapted for, rather than large ecological niches^41^. Furthermore, locomotor costs can also be important factors in small vertebrates. Given their ecological niche, locomotor economy may represent a significant constraint for the evolution of lizards.

## Supplementary information

Dataset 1

Dataset 2

## Data Availability

All the data used in the statistical tests can be found in Supplementary Material (Dataset 2).
