# Supplementary material for: Small vertebrates running on uneven terrain: a biomechanical study of two differently specialised lacertid lizards
Source: Sci Rep. 2019 Nov 14;9:16858. doi: 10.1038/s41598-019-53329-5 (PMC6856151; doi:10.1038/s41598-019-53329-5)
Supplement: Supplementary file 1 — Dataset 1 [file 41598_2019_53329_MOESM1_ESM.docx]

**Small vertebrates running on uneven terrain: a biomechanical study of two differently specialised** **lacertid lizards**

François Druelle^1*^, Jana Goyens^1^, Menelia Vasilopoulou-Kampitsi^1^, Peter Aerts^1,2^

^1^Laboratory for Functional Morphology, University of Antwerp, Belgium

^2^Department of Sport Sciences, University of Ghent, Belgium

*Corresponding author: [francois.druelle@uantwerpen.be](mailto:francois.druelle@uantwerpen.be)

**Supplementary material**


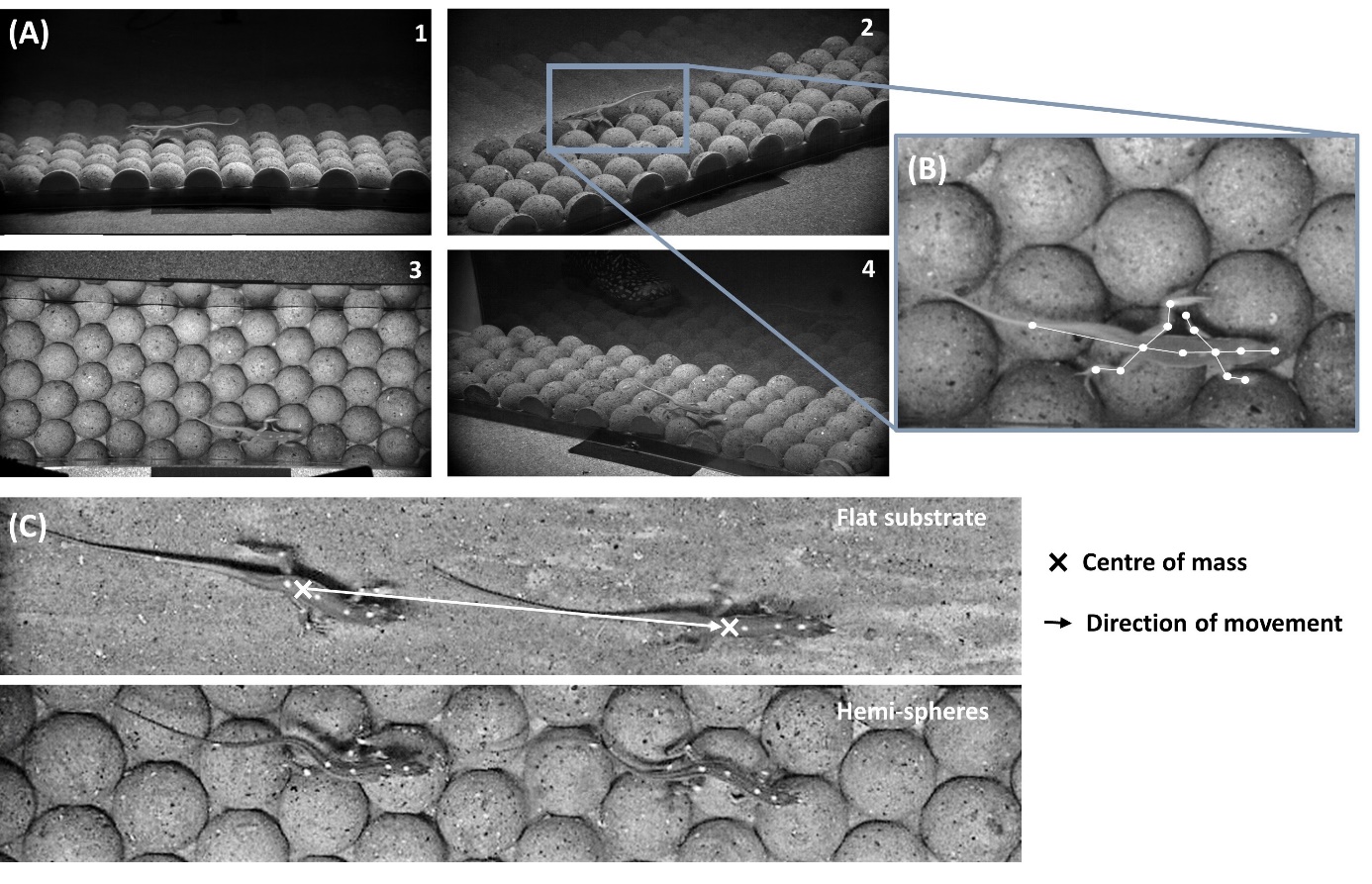


**Figure A.** Overview of the running experiments. (A) 4 views (1. Lateral, 2. Diagonal left side, 3. Top, 4. Diagonal right side) obtained from the high-speed video cameras. (B) Zoom-in view of the top view including the highlighted markers. (C) Top views of an *A. boskianus* individual running on the even and uneven substrates including the estimated position of the centre of mass and the direction of movement used in the rotation matrix (see method).

| **TABLE A. Mean ± SD for morphological variables** | | | | | | |
| --- | --- | --- | --- | --- | --- | --- |
|  | *A. boskianus* | | *P. muralis* | | Permutation | *P value* |
|  | **Mean** | **SD** | **Mean** | **SD** |  |  |
| **Morphometrics (n=7 *A. boskianus;* n=7 *P. muralis*)** | | | | | | |
| Body Mass (g) | 5.81 | 0.92 | 4.43 | 0.57 | 2.057 | ***0.0303*** |
| SVL^1^ (mm) | 63.95 | 3.18 | 61.26 | 3.19 | 1.176 | *0.268* |
| Tail length (mm) | 114.89 | 15.16 | 80.21 | 15.11 | 2.505 | ***0.007*** |
| Humerus length (mm) | 7.09 | 0.77 | 6.34 | 0.62 | 1.585 | *0.12* |
| Antebrachium (mm) | 7.96 | 0.46 | 6.25 | 0.38 | 2.884 | ***0.0029*** |
| Femur length (mm) | 11.18 | 0.91 | 8.29 | 0.83 | 2.896 | ***0.001*** |
| Tibia length (mm) | 13.37 | 1.54 | 8.60 | 0.90 | 3.024 | ***<0.0001*** |
| **Segment masses in % (n=3 *A. boskianus;* n=1 *P. muralis*)** | | | | | | |
| Head mass | 18.2 | 0.96 | 16.7 | N/A | - | - |
| Shoulder mass | 15 | 0.74 | 13.1 | N/A | - | - |
| Mid trunk mass | 17.3 | 0.13 | 15.1 | N/A | - | - |
| Hip mass | 16.4 | 1.82 | 21.4 | N/A | - | - |
| Tail mass | 17.5 | 1.28 | 23.6 | N/A | - | - |
| Forelimb mass | 2.2 | 0.05 | 1.8 | N/A | - | - |
| Hind limb mass | 5.5 | 0.13 | 2.3 | N/A | - | - |
| ^1^Snout Vent Length; numbers in bold indicate significant differences | | | | | | |
